# Theoretical Analysis of Enhanced Microwave Measurement Using Structured Beams

**DOI:** 10.3390/s26061966

**Published:** 2026-03-21

**Authors:** Zheng Yin, Feng Gao, Tianyu Chen, Chenxu Wang, Xiao Lu, Aihong Yang, Yandong Peng

**Affiliations:** 1 Qingdao Key Laboratory of Terahertz Technology, College of Electronic and Information Engineering, Qingdao 266590, China; yzzwo1@163.com (Z.Y.);; 2 College of Electrical Engineering and Automation, Shandong University of Science and Technology, Qingdao 266590, China

**Keywords:** microwave sensing, Bessel–Gaussian beams, Rydberg atoms

## Abstract

A theoretical scheme for precise measurement of microwave (MW) electric fields is proposed using a structured control field in Rydberg atoms. We use a Bessel–Gauss (BG) beam to drive the excited-state transition, its spatial structure characteristics result in a narrow linewidth of probe transmission, which benefits MW electric field measurement. It is interesting that the spectral linewidth could be further narrowed by increasing the azimuthal index. The minimum detectability of the MW field is about one-tenth of the common electromagnetically induced transparency scheme, and the spectrum resolution could be improved by about 40 times from simulation. Moreover, the system has good robustness.

## 1. Introduction

Rydberg-atom antennas have attracted increasing amounts of interest in the fields of quantum radars and novel microwave (MW) communications due to their high sensitivity to MW fields [1,2,3,4,5,6,7,8]. The properties of Rydberg atoms, e.g., their self-calibration and SI-traceable nature, make them ideal candidates for weak MW field detection [9]. The atomic antennas are mainly based on electromagnetically induced transparency (EIT) and Autler–Townes (AT) splitting techniques [10,11,12]. Recent research works include superheterodyne detection [13], cavity-enhanced sensing [14,15], MW vector detection [16,17,18,19,20,21] and continuous-frequency MW detection [22,23,24], etc. Most of these studies focused on the sensitivity or detection range of MW sensing, while a few researchers have addressed the influence of coupling beams’ spatial structure.

It is known that Laguerre–Gaussian (LG) and Bessel–Gaussian (BG) beams are two important light structures that have been widely used in optical communications and optical microscopy, etc. They show some advantages in precise measurement and detailed analysis [25,26]. The spatial intensity distribution of LG beams has been studied. It is shown that an LG beam can narrow the spectral linewidth and improve EIT spectrum [27,28,29]. BG beams are formed of a combination of non-diffracting Bessel beams and Gaussian beams, and they have been studied in turbulent environments [30,31,32].

In this work, we proposed a scheme for MW electric field measurement with Rydberg atoms driven by BG beams. The probe and BG beams counter-propagate through the atomic medium, and the spatial structure characteristics of the BG beam induce the inhomogeneous intensity distribution at the transverse cross-section of the beam and then reduce the effective intensity of the control field, which narrows the linewidth of the probe transmission. Moreover, increasing the azimuthal index *l* could further narrow the linewidth. The minimum detectability of MW field is about one-tenth of the common EIT scheme, and its linewidth reduces to 1/40 of the common EIT scheme, which means the measurement resolution could be improved by about 40 times. The numerical results are based on simulations.

It is worth noting that both [14] and our scheme compare their linewidths with the common EIT scheme, but they have different physical mechanisms. Under the same condition, ref. [14] showed the narrow linewidth induced by cavity coupling, while, here, we discussed MW measurement using a special beam. The BG beam has spatial redistribution, which changes the coherence interaction region between light and atoms. Thus, the effective Rabi frequency of the BG scheme improves the probe transmission. MW electric field measurement based on a BG scheme also has a better performance than an LG beam scheme. Moreover, this scheme shows good robustness, which may help with the design of MW sensing devices.

## 2. Theoretical Model

**Figure 1:**
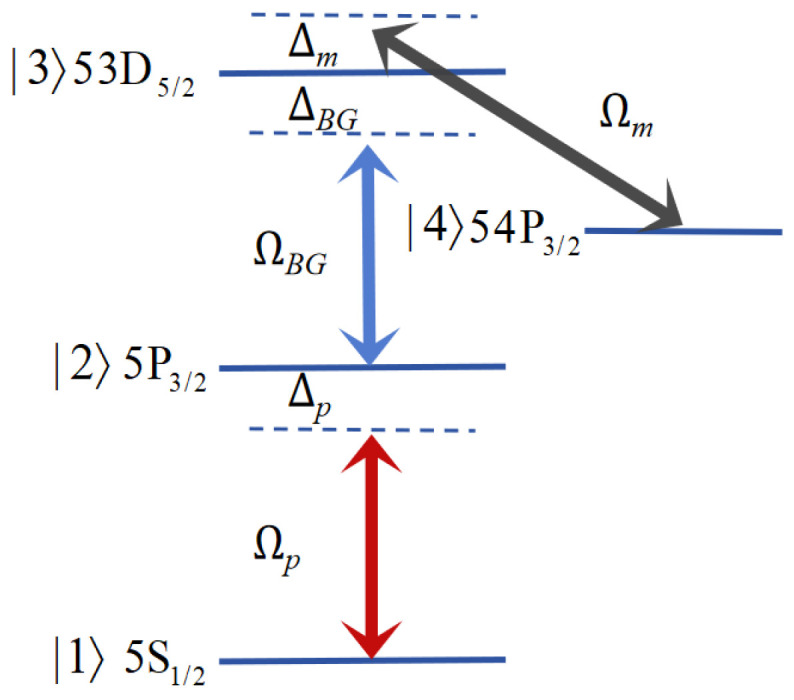
Four-level Rydberg atom model.

A four-level Rydberg atom system is considered [11], as shown in Figure 1. A Gaussian probe field $\Omega_{p}$ drives the transition between the states |1〉 and |2〉. A BG control field $\Omega_{BG}$ drives the transition between the states |2〉 and |3〉, which is different from the common EIT scheme with a Gaussian beam. The MW field $\Omega_{m}$ couples the Rydberg states |3〉 and |4〉.

In the interaction picture and after the rotating wave approximation, the Hamiltonian of the system can be derived as $H=-\hbar [\Delta_{p}|2\rangle \langle 2|+\left(\Delta_{p}+\Delta_{c}\right)\left|3\rangle \langle 3\right|+\left(\Delta_{p}+\Delta_{BG}+\Delta_{m}\right)\left|4\rangle \langle 4\right|+\Omega_{p}|1\rangle \langle 2|\;+\Omega_{BG}\left|2\rangle \langle 3\right|+\Omega_{m}|3\rangle \langle 4|+H.C.]$, where $\Omega_{p}=\mu_{12}E_{p}/\hbar$, $\Omega_{BG}=\mu_{12}E_{BG}/\hbar$ and $\Omega_{m}=\mu_{34}E_{m}/\hbar$ represent the Rabi frequency of the probe, control and MW fields, respectively. $\Delta_{p}$, $\Delta_{BG}$ and $\Delta_{m}$ are the detunings of the corresponding fields.

The dynamic process of the system can be described by the density-matrix method, $\dot{\rho }=-\frac{i}{\hbar }\left[H,\rho \right]+L\left(\rho \right)$ [33]. We assume that our probe field is much weaker than the control field, and the initial conditions are $\rho_{11}^{\left(0\right)}\approx 1$ and $\rho_{jj}^{\left(0\right)}\approx 0$ (*j* = 2, 3, 4). The atomic susceptibility for the probe field is given by:

(1) $$\begin{array}{c} \chi =\frac{2N{\left|\mu_{12}\right|}^{2}}{\varepsilon_{0}\hbar }\frac{-i[-\left(\gamma_{31}+i\Delta_{1}\right)\left(\gamma_{41}+i\Delta_{2}\right)-\Omega_{m}^{2}]}{\left(\gamma_{41}+i\Delta_{2}\right)\Omega_{BG}^{2}-\left(\gamma_{21}-i\Delta_{p}\right)\left[-\left(\gamma_{31}+i\Delta_{1}\right)\left(\gamma_{41}+i\Delta_{2}\right)-\Omega_{m}^{2}\right]}, \end{array}$$

where *N* is the atomic density. $\gamma_{jk}=\left(\Gamma_{k}+\Gamma_{j}\right)/2$ (*j*, *k* = 1, 2, 3, 4), $\Gamma_{k}$ represents the decay rate of state |k〉. $\Delta_{1}=\Delta_{p}+\Delta_{BG}$ and $\Delta_{2}=\Delta_{p}+\Delta_{BG}-\Delta_{m}$. The medium transmission can be expressed as T=exp−2πLIm[χ]/λp [34], where *L* is the medium length, and [34], where *L* is the medium length, and $\lambda_{p}$ is the wavelength of the probe laser.

In our model, the probe beam is considered as a Gaussian mode under the plane-wave approximation, whose Rabi frequency is a constant. Meanwhile, our control beam $\Omega_{BG}$ has inhomogeneous intensity distribution on a transverse plane, so the control field can be expressed as [30] $E\left(r,\varphi \right)=AJ_{l}\left(k_{r}r\right)\exp\left(il\varphi \right)\exp\left(-\frac{r^{2}}{\omega_{0}^{2}}\right)$, where *A* is the amplitude of the control field. $J_{l}$ denotes the Bessel function of the first kind of order *l*, where *l* is also the azimuthal index of BG mode, kr is the radial wave vector and *r* is the radius from the z-axis. *r* and φ are the polar coordinates and $\omega_{0}$ is a positive constant of the Gaussian term. To obtain the atomic susceptibility, a numerical integration over *r* is considered. And the Rabi frequency of the control field yields that

(2) $$\Omega_{BG}=\int_{0}^{r}\left|\Omega_{c}J_{l}\left(k_{r}r\right)\exp\left(il\varphi \right)\exp\left(-\frac{r^{2}}{\omega_{0}^{2}}\right)\right|dr,$$

where $\Omega_{c}$ is the Rabi frequency of a common EIT control field.

## 3. Results and Discussion

**Figure 2:**
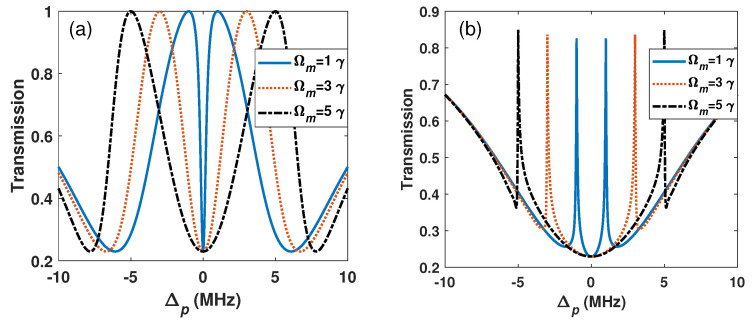
(**a**) The common EIT transmission and (**b**) the EIT transmission with BG beam for different MW fields $\Omega_{m}$. $\Omega_{c}=6\;\gamma$, $\Delta_{BG}=\Delta_{m}=0$, $\gamma_{31}=0.001\;\gamma$, $\gamma_{41}=0.0005\;\gamma$ and l=1.

First, we consider the numerical result of a common EIT transmission, as shown in Figure 2a. The peak-to-peak distance changes linearly with the MW field, which can be used to measure the MW field. For simplicity, the parameters are scaled by $\Gamma_{2}=6\;\gamma =2\pi \times 6\;\mathrm{MHz}$ in the following discussion. Then, in our scheme, the control field is considered as BG beam (Equation (2)), and this beam is aligned to the centre of the probe beam in an atomic vapour cell, and the beam radius of the interaction region is about 1.2 mm. The probe transmission is shown in Figure 2b. The spectrum shows a narrower linewidth compared with the common EIT scheme, which benefits the MW electric field measurement. The numerical results show that the linewidth of the common EIT transmission is about 3.11 γ, while the linewidth of the BG scheme is only 0.094 γ under the condition of $\Omega_{m}=\gamma$. The narrow linewidth of our scheme is due to the BG mode of the control field. The vortex structure at the center and the characteristic of the Bessel function-like distribution indicate that the BG beam has an inhomogeneous intensity distribution at the transverse section of the beam [29]. The inhomogenenity in the BG beam changes the coherence interaction region between light and atoms, which reduces the effective intensity of the control field. As a result, the spectral linewidth narrows significantly. It is noted that the Bessel function-like distribution at the transverse section of the BG beam further narrows the linewidth of the spectrum. This feature of the BG scheme may benefit MW measurement [29].

**Figure 3:**
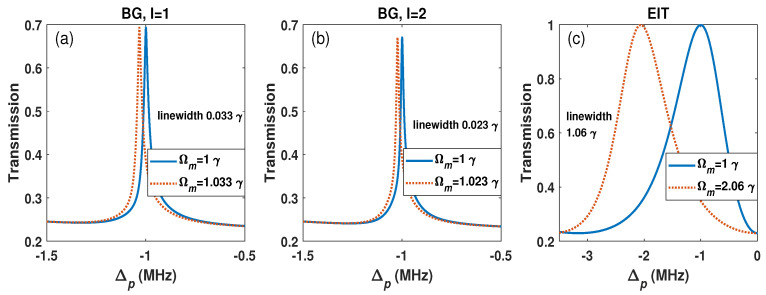
The transmission of the BG scheme with (**a**) l=1 and (**b**) l=2, and (**c**) the common EIT transmission. $\Omega_{c}=3\;\gamma$. The other parameters are the same as in Figure 2.

MW electric field measurement cares about the spectrum resolution. According to the Rayleigh criterion, the spectrum resolution is related to the linewidth, i.e., the full width at half maximum (FWHM). A narrow transmission peak indicates a high spectrum resolution. The numerical result shows that the FWHM of the BG scheme is about 0.033 γ under the condition of $\Omega_{m}=\gamma$ and $\Omega_{c}=3\;\gamma$, as shown in Figure 3a. It is interesting to find that the BG beam with a large azimuthal index *l* could narrow the FWHM, further improving the spectrum resolution. For example, when l=2, the FWHM of the probe transmission is about 0.023 γ from simulation, and this linewidth is narrower than the linewidth of 0.033 γ with l=1, as shown in Figure 3a,b. This is because the azimuthal index *l* changes the intensity distribution at the transverse section of the beam. For a large *l*, the vortex size at the center becomes large, causing the main beam intensity distribution to shift from the center to the outside. Meanwhile, too large *l* will weaken the EIT peak. For example, when l>2, the simulation result shows that the EIT peaks of the BG scheme reduce to half, which does not benefit MW measurement. Here, we consider l=2 in the following discussion. Figure 3c shows the linewidth of the common EIT under the condition of $\Omega_{m}=\gamma$ and $\Omega_{c}=3\;\gamma$. The linewidth of the common EIT scheme is about 1.06 γ, while the BG scheme is only 0.023 γ, which indicates that the linewidth of the BG scheme is much narrower than that of the common EIT scheme. The numerical results show that the spectrum resolution of the BG scheme could be improved by about 40 times compared with the common EIT scheme.

**Figure 4:**
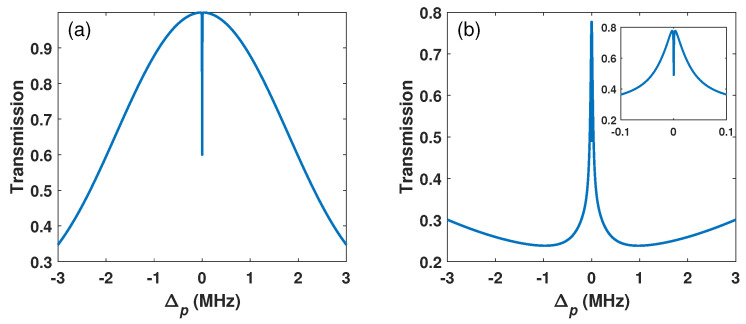
(**a**) The common EIT transmission and (**b**) EIT spectrum with BG beam for MW field $\Omega_{m}=0.03\;\gamma$ and $\Omega_{m}=0.0027\;\gamma$, respectively (inset is zoomed view of central peak). $\Omega_{c}=4.5\;\gamma$, l=2. The other parameters are the same as in Figure 2.

The minimum detectable MW field is another important index for the MW electric field measurement. According to the Rayleigh criterion, the minimum detectable strength depends on the minimum detectable splitting, i.e., the splitting of two transmission peaks is about half the maximum of its peak value. The numerical results show that the minimum detectable MW field strength for the common EIT scheme is about 0.03 γ, as shown in Figure 4a. Meanwhile, the minimum detectable MW field strength of the BG scheme is only 0.0027 γ (see Figure 4b), which is about one-tenth of the common EIT scheme. The small minimum detectable MW field strength of the BG scheme is attributed to its narrow spectral characteristics, where the double EIT peaks show a clear split, as shown in the inset of Figure 4b.

**Figure 5:**
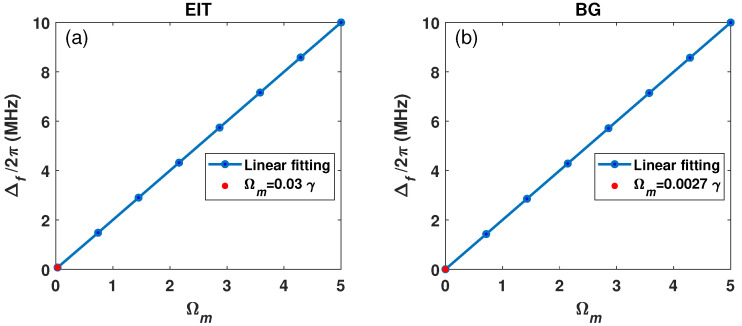
(**a**) The transmission peak intervals of common EIT and (**b**) the EIT with BG beam for different MW fields and their linear fitting. $\Omega_{c}=4.5\;\gamma$, l=2. The other parameters are the same as in Figure 2.

Figure 5 shows the transmission peak intervals of common EIT and BG schemes for different MW field strengths. The peak splitting intervals of the BG scheme show linear relationship with MW field, as seen in the common EIT scheme. The numerical results show that the BG beam does not change the EIT-AT splitting interval.

**Figure 6:**
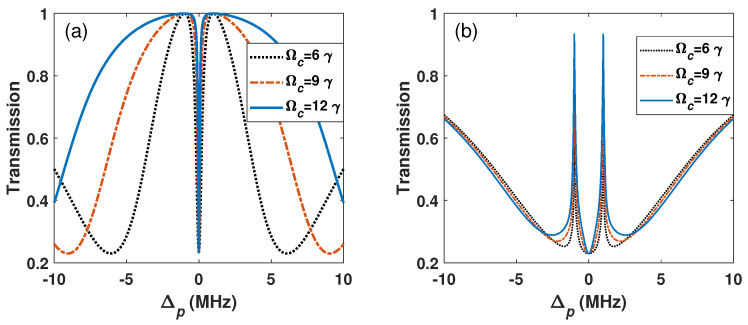
(**a**) The common EIT transmission and (**b**) the EIT spectrum with the BG beam for different $\Omega_{c}$. $\Omega_{m}=1\;\gamma$, l=2. The other parameters are the same as in Figure 2.

Figure 6 shows the probe transmission of the common EIT and BG scheme with different control field strength, where the intensity of BG beam $\Omega_{BG}$ is a function of $\Omega_{c}$ in Equation (2). The linewidth of the common EIT scheme becomes broad as the control field strength increases, as shown in Figure 6a. Meanwhile, the linewidth of the BG scheme remains a narrow characteristic (see Figure 6b), which means that the BG scheme seems to be robust against control field perturbations. For example, when $\Omega_{c}$ increases from 6 γ to 12 γ, the linewidth of the common EIT scheme increases by 5.39 γ, and that of the BG scheme increases by 0.13 γ. These numerical results show that the linewidth increases shown for the BG scheme are about 1/40 of those in the common EIT scheme. This means that the BG scheme shows good robustness to the driving field fluctuation and retains a high sensitivity of MW measurement for strong driving fields.

**Figure 7:**
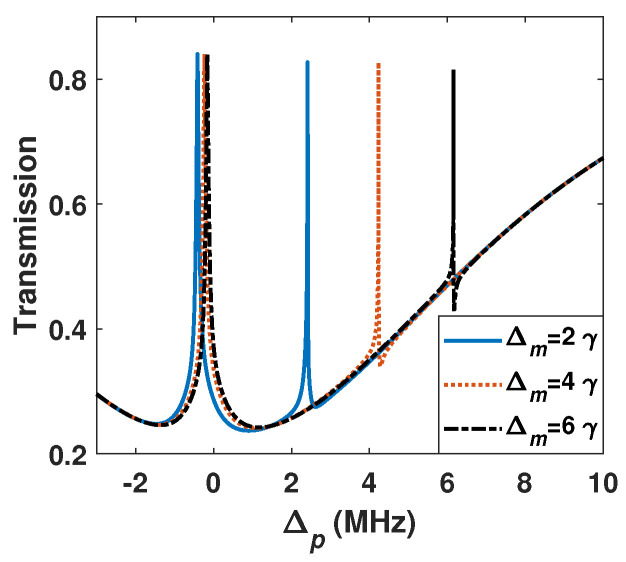
EIT spectrum with BG beam for different MW field detunings $\Delta_{m}$. l=2. The other parameters are the same as in Figure 2.

At last, we consider the effect of frequency detunings. Figure 7 shows the probe transmission with different MW field detunings $\Delta_{m}$. The numerical results show that the MW detunings broaden the peak-to-peak distance compared to the resonance one. For example, when $\Delta_{m}$ = 0 γ, the peak-to-peak distance is 2 γ, when $\Delta_{m}$ = 2 and 4 γ, the peak-to-peak distance is about 2.83 and 4.47 γ, respectively. The peak separation $\Delta f_{m}$ and MW field detunings $\Delta_{m}$ satisfy $\Delta f_{m}={\left(\Delta_{m}^{2}+\Delta f^{2}\right)}^{1/2}$ [34], where Δf is the peak-to-peak distance with MW on resonance. So, some MW field detunings broaden the peak-to-peak distance, which helps with weak MW detection. Of course, we notice that the peak values of the two transmission peaks are different from each other, which is consistent with a recent report [35].

Here, we summarize the comparisons of our proposed scheme and common EIT scheme. In our scheme, the spectrum resolution is about 40 times that of the common EIT scheme, and the minimum detectable MW field is about one-tenth of the common EIT scheme. In addition, our scheme shows better robustness compared with the common EIT schemes.

In experiments, the parameters may be based on those reported in Rydberg–EIT experiments [11]. The MW measurement is based on 87Rb Rydberg atoms and the 780 nm probe beam drives the ground-state transition, $5S_{1/2}$ (F = 2)-$5P_{3/2}$ (F = 3), whereas the 480 nm control beam drives the excited-state transition, $5P_{3/2}$ (F = 3)-$53D_{5/2}$ (F = 4) and the MW field drives the Rydberg-state transition, $53D_{5/2}$ (F = 4)-$54P_{3/2}$ (F = 3). The Rb vapour cell length is about 0.05 m. The BG control beam could be generated from the Gaussian-type control beam with a spatial light modulator via computer-generated holograms, and BG modes are imaged using a CCD camera [31]. This beam is aligned to the centre of the probe beam in an atomic vapour cell, and the beam radius of the interaction region is about 1.2 mm. The radial wave vector is approximately $5\times 10^{3}\;\mathrm{rad}/m$. Then, the probe transmission is collected by a detector.

## 4. Conclusions

In summary, a theoretical scheme for measuring the MW electric field is proposed using a spatial structure beam to drive the excited-state transition in a Rydberg-atom system. The BG beam has an inhomogeneous intensity and Bessel function-like distribution, which changes the coherence interaction region between the laser beams and atoms and shows a narrow spectrum linewidth. The linewidth can be further narrowed by increasing the azimuthal index *l*. The minimum detectability of the MW field is about one-tenth of the common EIT scheme, and the spectrum resolution could be improved by about 40 times from simulation. Moreover, the BG scheme shows better robustness than that of the common EIT scheme. The scheme may be useful for designing MW sensing devices. 

## Data Availability

Some or all data that support the findings of this study are available from the corresponding author upon reasonable request.
